# Virtual reality in Parkinson’s disease: a systematic review and meta-analysis

**DOI:** 10.1590/1980-5764-DN-2024-0257

**Published:** 2025-09-19

**Authors:** João Vitor Andrade Fernandes, Vera Louise Freire de Albuquerque Figueiredo, Afonso Bezerra Oliveira, Isabelle Albuquerque Reis, Gabrielle de Lacerda Dantas Henrique, Estácio Amaro da Silva

**Affiliations:** 1 Universidade Federal da Paraíba, João Pessoa PB, Brazil.

**Keywords:** Virtual Reality, Parkinson Disease, Systematic Review, Meta-Analysis, Realidade Virtual, Doença de Parkinson, Revisão Sistemática, Metanálise

## Abstract

Parkinson’s disease (PD) severely affects motor and non-motor functions, leading to dependency and reduced quality of life. Conventional rehabilitation methods often fail to meet patients’ diverse needs. Virtual reality (VR) offers an innovative approach by creating immersive, customizable therapeutic environments.

**Objective:**

This systematic review and meta-analysis evaluated the efficacy of VR interventions in PD rehabilitation compared to conventional therapies.

**Methods:**

Following the Preferred Reporting Items for Systematic Reviews and Meta-Analyses (PRISMA) guidelines, a systematic search was conducted across five databases for randomized controlled trials (RCTs) published until November 2023. Inclusion criteria encompassed RCTs comparing VR with other interventions in PD patients. Data on motor and balance outcomes were extracted. Risk of bias was assessed using the RoB 2 tool.

**Results:**

Five RCTs, including 199 participants, were analyzed. VR interventions demonstrated significant improvements in the Time Up-to-Go (TUG) test (mean difference: -2.42; 95% confidence interval – 95%CI -3.95 to -0.89; p=0.002), indicating enhanced dynamic balance. However, the Berg Balance Scale (BBS) results favored the control group (mean difference: 3.28; 95%CI 1.92 to 4.65; p<0.00001).

**Conclusion:**

VR interventions significantly improve dynamic balance and mobility in PD patients, as evidenced by TUG results. The limited impact on static balance tasks highlights the need for tailored interventions. While VR shows promise as a complementary therapy, challenges such as cost, accessibility, and standardization must be addressed to enhance its clinical utility.

## RESUMO.

A doença de Parkinson (DP) compromete funções motoras e não motoras, aumentando a dependência e reduzindo a qualidade de vida. Métodos convencionais de reabilitação frequentemente não atendem às necessidades dos pacientes. A realidade virtual (RV) apresenta uma abordagem inovadora, criando ambientes terapêuticos imersivos e personalizados.

**Objetivo:**

Avaliar a eficácia das intervenções com RV na reabilitação da DP em comparação com terapias convencionais.

**Métodos:**

Seguindo as diretrizes *Preferred Reporting Items for Systematic Reviews and Meta-Analyses* (PRISMA), foi realizada uma busca sistemática em cinco bases de dados para ensaios clínicos randomizados (ECR) publicados até novembro de 2023. ECR que comparavam RV com outras intervenções em pacientes com DP foram incluídos. Dados sobre desfechos motores e de equilíbrio foram extraídos. O risco de viés foi avaliado usando a ferramenta RoB2.

**Resultados:**

Cinco ECR, com 199 participantes, foram analisados. As intervenções com RV mostraram melhorias significativas no teste Time Up-to Go (TUG) (diferença média: -2,42; intervalo de confiança de 95% — IC95% -3,95 a -0,89; p=0,002), indicando melhora no equilíbrio dinâmico. No entanto, os resultados da Escala de Equilíbrio de Berg (BBS) favoreceram o grupo controle (diferença média: 3,28; IC95% 1,92 a 4,65; p<0,00001).

**Conclusão:**

As intervenções com RV melhoram significativamente o equilíbrio dinâmico e a mobilidade em pacientes com DP, conforme evidenciado pelos resultados do TUG. O impacto limitado nas tarefas de equilíbrio estático destaca a necessidade de intervenções personalizadas. Apesar do potencial da RV como terapia complementar, desafios como custo, acessibilidade e padronização precisam ser superados para ampliar sua aplicabilidade clínica.

## INTRODUCTION

 Parkinson’s disease (PD) is a progressive neurodegenerative disorder characterized by motor symptoms, including bradykinesia, rigidity, resting tremor, and postural instability, as well as non-motor manifestations such as cognitive decline, mood disturbances, and autonomic dysfunction^1-3^. These symptoms lead to increased dependency and incapacity, making daily tasks challenging for those affected. While conventional rehabilitation strategies provide symptomatic relief, they often fail to comprehensively address the complex motor and cognitive impairments in PD, necessitating the development of adjunctive therapeutic modalities^4^. 

 Virtual reality (VR) has recently garnered attention as a cutting-edge approach for neurological rehabilitation. VR provides an engaging, dynamic, and customizable environment that can simulate real-life scenarios, offering a unique and controlled setting for therapeutic activities^5^. This immersive technology allows for the creation of tailored therapies that can adapt to the individual needs of patients, enhancing motivation and adherence to treatment regimens. By incorporating VR into rehabilitation programs, therapists can offer more effective and personalized interventions, potentially improving overall treatment efficacy^6-8^. VR systems are categorized based on immersion levels: non-immersive VR utilizes conventional screens and input devices; semi-immersive VR employs large-screen projections or limited head-mounted displays; and fully immersive VR integrates advanced head-mounted displays with motion tracking for a fully interactive experience^9,10^. The degree of immersion may influence neuroplasticity and therapeutic efficacy, highlighting the need for systematic evaluation of VR applications in PD rehabilitation^11,12^. 

 Recent studies support the efficacy of VR-based interventions in PD rehabilitation, demonstrating significant improvements in motor function, postural stability, and fall risk reduction^13-15^. To consolidate these findings, this systematic review and meta-analysis aim to bridge this knowledge gap by compiling current data on the effects of VR therapy in PD patients. The review evaluates VR therapy’s effectiveness for PD patients compared to conventional approaches. 

## METHODS

### Protocol and registration

 This systematic review strictly followed the Preferred Reporting Item for Systematic Reviews and Meta-Analyses (PRISMA) guidelines and was previously registered on the International Prospective Register of Systematic Reviews (PROSPERO) International prospective register of systematic reviews (CRD42023420596)^16^. 

### Search strategy and inclusion criteria

 Two independent authors (JVAF and ABON) conducted a systematic search in United States National Library of Medicine (PubMed), the Cochrane Library, Embase, Web of Science, and Science Direct for studies published from inception to November 2023. The search terms included "virtual reality," "neurological rehabilitation," and "randomized controlled trial." The detailed search strategy for each database is provided in Table 1. We included studies that met the following criteria:

- randomized controlled trials;
- comparing VR with other interventions; and
- involving patients diagnosed with PD.

**Table 1:** Search strategy across databases. This table summarizes the search strategies used in each database to identify relevant randomized controlled trials for the systematic review.

Database	Search Strategy
PubMed	"Virtual Reality" [Mesh] AND "Neurological Rehabilitation" [Mesh] AND random*
Cochrane	("Virtual Reality"[Mesh]) AND "Neurological Rehabilitation"[Mesh]
Web of Science	(Realidade Virtual OR Realidade Virtual Educativa OR Realidade Virtual Instrucional OR Realidades Virtuais OR Realidades Virtuais Educativas OR Realidades Virtuais Instrucionais OR Educational Virtual Realities OR Educational Virtual Reality OR Instructional Virtual Realities OR Instructional Virtual Reality OR Realities, Instructional Virtual OR Reality, Educational Virtual OR Reality, Instructional Virtual OR Reality, Virtual OR Virtual Realities, Educational OR Virtual Realities, Instructional OR Virtual Reality, Educational OR Virtual Reality, Instructional OR Virtual Reality OR Realidad Virtual OR Réalité de synthèse) AND (Reabilitação Neurológica OR Neurological Rehabilitation OR Rehabilitación Neurológica OR Rééducation neurologique)
ScienceDirect	"Virtual Reality" AND "Neurological Rehabilitation"
Embase	‘virtual reality’/syn AND ‘neurorehabilitation’/syn AND ‘randomized controlled trial’/syn

 Exclusion criteria were:

- non-randomized studies;
- nonhuman studies; and
- studies lacking standardized measures for PD.

There were no restrictions on language or publication year. After the initial search, two authors independently removed duplicates, screened titles and abstracts, and assessed articles for eligibility. Discrepancies were resolved through consensus-based discussion. 

### Data extraction and outcomes

 Data were primarily sourced from published articles, with additional individual patient data provided by the authors when available. The collected data encompassed population baseline characteristics, details of each treatment, and the definitions and time frames of the outcomes. The outcomes assessed included changes in clinical symptoms, specifically motor skills and balance. Rayyan software was used to facilitate study selection and screening in accordance with predefined eligibility criteria. 

### Statistical analysis and risk of bias assessment

 Means and standardized differences were collected both before and after interventions across studies to facilitate meta-analyses of commonly assessed outcomes. A 95% confidence interval was applied to these measures. Statistical analyses were conducted using R version 4.3.1 software. Two independent authors (JVAF and ABON) used the Cochrane Collaboration Risk of Bias tool version 2 (RoB 2) to assess the risk of bias in each individual RCT^17^. 

## RESULTS

### Search results

**Figure 1:**
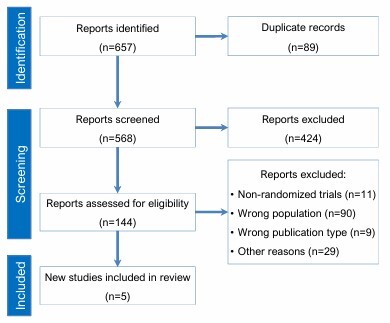
Flow diagram illustrating the study selection process for the systematic review and meta-analysis, following the Preferred Reporting Items for Systematic Reviews and Meta-Analyses (PRISMA) guidelines.

 As detailed in Figure 1, our initial search yielded 657 studies. After duplicates and non-related studies were removed, 149 articles remained and were assessed for full-text review according to the eligibility criteria. Of those, five randomized controlled trials were included, comprising 199 participants, 124 men and 75 women^18-22^. All trials took place between 2014 and 2019. A comprehensive description of the study characteristic can be found in Table 2. 

**Table 2:** Characteristics of included studies. This table provides detailed information about the included studies, including their country of origin, study population, cognitive function (assessed by the Mini-Mental State Examination), disease severity (Hoehn & Yahr stages), intervention, and control group characteristics.

Study	Country	Population	MMSE Scores	Hoehn & Yahr Stages	Intervention	Control
Pazzaglia et al.^18^	Italy	51 patients (16 female) with PD. (Mean age = 71 years).	>25	Not specified	Semi-immersive VR (n=25).	Conventional rehabilitation programme (n=26).
Ribas et al.^19^	Brazil	20 patients (8 female) with PD. (Mean age = 61 years).	≥24	I–III	Non-immersive VR (n=10).	Conventional exercise (n=10).
Feng et al.^20^	China	28 patients (13 female) with PD. (Mean age = 67.2 years).	Not reported	2.5–4*	Semi-immersive VR (n=14).	Conventional physical therapy (n=14).
Gandolfi et al.^21^	Italy	76 patients (25 female) with PD. (Mean age = 68.6 years).	≥24	2.5–3*	Non-immersive VR (n = 38)	In-clinic sensory integration balance training (n = 38)
Liao et al.^22^	Taiwan	24 patients (13 female) with PD. (Mean age = 64.85 years).	≥24	I–III	Semi-immersive VR + Treadmill training (n=12).	Traditional exercise + Treadmill training (n=12)

Abbreviations: MMSE, Mini-Mental State Examination; PD, Parkinson’s Disease; VR, Virtual Reality; n Number of Participants.

Note: *Indicates the use of the Improved Hoehn-Yahr classification.

### Virtual reality interventions

 As seen in Table 2, the five studies that met the eligibility criteria evaluated different interventions of the use of virtual reality and their effects in patients with Parkinson’s disease. These included: VR rehabilitation programme, Exergaming, VR training, VR balance telerehabilitation and VR-based Wii Fit exercises in association of treadmill training. The duration of the intervention periods analyzed ranged from six to 12 weeks, the frequency of the sessions ranged from two to five times per week, with an average of 25.8 sessions, with each session lasting between 30 and 60 minutes. 

### Outcome measures effects

#### Berg Balance Scale (BBS)

**Figure 2:**
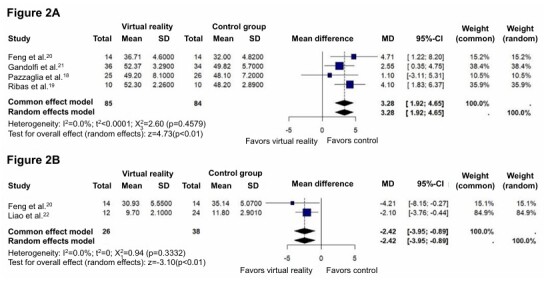
Effect of virtual reality interventions on balance and mobility in Parkinson’s disease. **(A)** Forest plot displaying the effect size and confidence intervals for studies evaluating the impact of virtual reality interventions on Berg Balance Scale (BBS) scores in Parkinson’s disease patients. **(B)** Forest plot showing the effect size and confidence intervals for studies assessing the impact of virtual reality interventions on Time Up-to-Go (TUG) test performance in Parkinson’s disease patients.

 Four studies were evaluated, involving 85 individuals in the intervention group and 84 in the comparison group. The mean difference found in the forest plot was 3.28, with a confidence interval of 1.92 to 4.65, indicating a strong trend favoring Control, with very strong statistical significance (p<0.00001). There was no heterogeneity among the studies (I^2^=0%), suggesting a high consistency in the results among studies. Overall, the effect size Z was 4.73, reinforcing the strong statistical significance for this outcome (Figure 2A). 

#### Time Up-to-Go (TUG) Test

 Two studies analyzed this outcome, involving 26 individuals in the intervention group and 38 in the control group. The mean difference obtained was -2.42, with a confidence interval of -3.95 to -0.89, favoring the VR group. The absence of heterogeneity (I^2^=0%) indicates high consistency in the study results. The effect size Z of 3.10 and the p-value of 0.002 indicates that the difference is statistically significant in favor of the experimental group, suggesting that VR interventions are more effective in improving balance as measured by the TUG compared to the control (Figure 2B). 

### Risk of bias assessment

**Figure 3:**
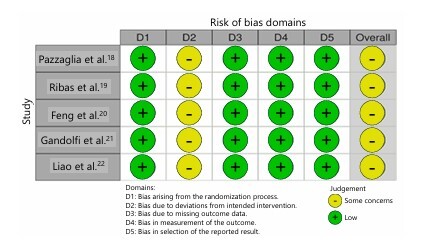
Risk of bias assessment. Summary of the risk of bias evaluation for the included randomized controlled trials, categorized by domain according to the Cochrane Risk of Bias 2 tool.

 The RoB-2 assessment identified five studies with some concerns (Figure 3). All studies raised some concerns regarding the participants being aware of their assigned intervention. All other domains were consistently identified as having a low risk of bias. 

## DISCUSSION

 This systematic review and meta-analysis examined the effectiveness of VR interventions in the neurological rehabilitation of PD. VR interventions demonstrated notable benefits in the TUG test, while the results of the BBS were negative. These findings underscore the potential of VR as a complementary tool for PD rehabilitation, though its efficacy may vary depending on the specific outcomes assessed. 

 VR offers a unique advantage in PD rehabilitation by simulating real-life scenarios in a controlled, adaptable environment. This immersive approach enables patients to practice motor and balance tasks in engaging, low-risk settings^23^. Clinically, VR could address key challenges faced by PD patients, such as impaired coordination and gait instability, by promoting neuroplasticity and enhancing motor learning through repeated practice^24,25^. Furthermore, VR’s customizable nature allows therapists to tailor interventions to individual patient needs, potentially increasing adherence and motivation — a common barrier in traditional rehabilitation programs^23^. 

 From a clinical perspective, the integration of VR into rehabilitation aligns with the multidimensional management of PD, complementing pharmacological and physical therapy approaches^25^. However, widespread adoption requires careful consideration of patient characteristics, including the stage of the disease, cognitive function, and physical ability, to maximize safety and efficacy^26^. Despite its promise, VR has limitations that may hinder its applicability, particularly for older adults, who constitute the majority of PD patients. The technological complexity of VR systems may pose a significant barrier to older individuals unfamiliar with digital interfaces, potentially reducing engagement and effectiveness^27-29^. Financial constraints also limit accessibility, as VR equipment and associated therapies can be prohibitively expensive for many patients and healthcare systems, particularly in low-resource settings^30,31^. 

 Furthermore, VR interventions require specialized training for healthcare providers, adding to implementation challenges^31^. The lack of standardization in VR protocols across studies further complicates the integration of these technologies into routine clinical practice, underscoring the need for consensus on optimal VR designs and application methods^30,31^. 

 The current physical therapy guidelines for PD primarily emphasize traditional rehabilitation approaches, including resistance training, aerobic exercise, and balance exercises, but there is increasing interest in the potential role of VR in clinical practice^32^. While VR is not yet widely incorporated into formal guidelines, emerging evidence supports its use as an adjunctive tool for balance and gait training. Some recent recommendations suggest that VR-based interventions may enhance motor learning and functional mobility, particularly when integrated with structured physical therapy programs^33-35^. However, further high-quality research is needed to establish standardized protocols, define patient selection criteria, and determine the long-term benefits of VR-based rehabilitation in PD management. 

 The cognitive status and disease severity of the participants included in the analyzed studies may influence the effectiveness of VR interventions. Most studies applied the Mini-Mental State Examination (MMSE) as a cognitive screening tool, with cutoff scores ranging from ≥24 to >25, ensuring that individuals with significant cognitive impairment were excluded. Regarding disease severity, studies primarily included participants classified within Hoehn and Yahr stages I to IV, with some focusing on early to moderate stages (I–III) and others extending to more advanced cases (2.5–4). These criteria suggest that the benefits observed in VR interventions are particularly applicable to individuals with preserved cognitive function and mild to moderate PD severity. Future research should investigate whether VR-based rehabilitation remains effective for individuals with more severe cognitive deficits or advanced disease stages, as these populations may have different therapeutic responses and accessibility challenges^36-38^. 

 The BBS is a widely used clinical tool designed to assess static balance through a series of functional tasks that measure an individual’s ability to maintain posture while sitting, standing, and performing weight shifts^39^. In contrast, the TUG test evaluates dynamic balance and functional mobility by measuring the time required for a patient to stand up from a seated position, walk a short distance, turn, and return to a seated position^40^. These assessments capture different aspects of postural control and movement, which may explain the differing effects of VR interventions observed in this review. The positive outcomes observed in the TUG test suggest that VR excels in improving dynamic balance and functional mobility, likely due to its ability to simulate real-world challenges that enhance reactive and anticipatory postural adjustments^41,42^. In contrast, the limited improvement in the BBS may reflect the scale’s focus on static balance tasks, which VR may not address as effectively. We hypothesize that this discrepancy arises from VR’s emphasis on dynamic, task-specific training, which aligns more closely with the demands of the TUG test but less so with the static components of the BBS. 

 The findings of this meta-analysis suggest that VR interventions hold significant potential for future applications in PD rehabilitation, particularly in improving functional mobility and dynamic balance. Given its capacity to simulate real-world motor challenges, VR may be integrated into structured rehabilitation protocols to enhance gait adaptability, postural control, and fall prevention strategies^43-45^. Beyond its role in structured therapy, VR has potential as an assistive tool to support independent rehabilitation and mobility in PD patients^24^. With advancements in sensor-based tracking and real-time feedback mechanisms, VR could be incorporated into home-based training models, allowing continuous rehabilitation outside clinical settings. The integration of wearable motion sensors and artificial intelligence could further refine task difficulty, providing personalized feedback to enhance motor learning and adherence to therapy^46^. For VR to be widely adopted as an assistive device, challenges related to accessibility, affordability, and patient usability must be addressed^47^. Additionally, interdisciplinary efforts are needed to standardize VR protocols and ensure their seamless integration into clinical practice and assistive rehabilitation technologies. 

 One of the key limitations of this review is the variability in VR protocols across the included studies, which hinders direct comparisons of outcomes. The diversity in intervention designs, ranging from different levels of immersion to varying session durations and therapy goals, may contribute to the heterogeneity observed in the results. While this variability allows for tailored approaches to rehabilitation, it also makes it challenging to draw definitive conclusions about the most effective VR parameters for PD management. Standardization of VR methodologies in future clinical trials is crucial to ensure consistency in outcome measurement and facilitate a more accurate assessment of its therapeutic efficacy in PD rehabilitation. The development of evidence-based guidelines for VR implementation in PD rehabilitation should consider factors such as immersion level, task complexity, and patient-specific characteristics to optimize its clinical benefits^5,48,49^. 

 Additionally, while this study highlights the promise of VR interventions, the long-term effects of such therapies remain unclear due to the limited follow-up periods reported in the included studies. Given the progressive nature of PD, future research should prioritize investigating the sustainability of VR-induced benefits over extended periods^50^. The duration of VR-related improvements in motor function, balance, and quality of life remains a crucial question, as shortterm gains may not necessarily translate into lasting functional benefits. Longitudinal studies assessing the retention of motor and cognitive improvements post-intervention, along with their impact on activities of daily living, would provide valuable insights into VR’s role as a long-term therapeutic strategy in PD management. Moreover, cost-effectiveness analyses should be conducted to determine whether continued access to VR-based therapy justifies its implementation in routine clinical practice. 

 The findings highlight the promise of VR as a complement to traditional therapies, but its broader implementation requires addressing technological and financial barriers, especially for older adults. VR interventions demonstrate potential as an innovative approach to PD rehabilitation, particularly in enhancing dynamic balance and mobility, as evidenced by improved TUG test results. However, their limited impact on static balance tasks, such as those measured by the BBS, underscores the need for tailored intervention designs. Future research should prioritize standardizing VR protocols, exploring cost-effective solutions, and assessing the integration of VR with other therapeutic modalities to optimize its clinical utility in PD management. 

## Data Availability

The datasets generated and/or analyzed during the current study are available from the corresponding author upon reasonable request.
